# The clinical, neuroanatomical, and neuropathologic phenotype of *TBK1*-associated frontotemporal dementia: A longitudinal case report

**DOI:** 10.1016/j.dadm.2016.10.003

**Published:** 2016-11-03

**Authors:** Carolin A.M. Koriath, Martina Bocchetta, Emilie Brotherhood, Ione O.C. Woollacott, Penny Norsworthy, Javier Simón-Sánchez, Cornelis Blauwendraat, Katrina M. Dick, Elizabeth Gordon, Sophie R. Harding, Nick C. Fox, Sebastian Crutch, Jason D. Warren, Tamas Revesz, Tammaryn Lashley, Simon Mead, Jonathan D. Rohrer

**Affiliations:** a Department of Neurodegenerative Disease, MRC Prion Unit, UCL Institute of Neurology, London, UK; b Department of Neurodegenerative Disease, Dementia Research Centre, UCL Institute of Neurology, London, UK; c Genetics and Epigenetics of Neurodegeneration, Hertie Institute for Clinical Brain Research (HIH), Tübingen, Germany; d Applied Genomics for Neurodegenerative Diseases, German Centre for Neurodegenerative Diseases (DZNE), Tübingen, Germany; e Queen Square Brain Bank for Neurological Disorders, Department of Molecular Neuroscience, UCL Institute of Neurology, University College London, London, UK

**Keywords:** TBK1, Neurogenetics, Neuropathology, Frontotemporal dementia

## Abstract

**Introduction:**

Mutations in the TANK-binding kinase 1 (*TBK1*) gene have recently been shown to cause frontotemporal dementia (FTD). However, the phenotype of *TBK1*-associated FTD is currently unclear.

**Methods:**

We performed a single case longitudinal study of a patient who was subsequently found to have a novel A705fs mutation in the *TBK1* gene. He was assessed annually over a 7-year period with a series of clinical, cognitive, and magnetic resonance imaging assessments. His brain underwent pathological examination at postmortem.

**Results:**

The patient presented at the age of 64 years with an 18-month history of personality change including increased rigidity and obsessiveness, apathy, loss of empathy, and development of a sweet tooth. His mother had developed progressive behavioral and cognitive impairment from the age of 57 years. Neuropsychometry revealed intact cognition at first assessment. Magnetic resonance imaging showed focal right temporal lobe atrophy. Over the next few years his behavioral problems progressed and he developed cognitive impairment, initially with anomia and prosopagnosia. Neurological examination remained normal throughout without any features of motor neurone disease. He died at the age of 72 years and postmortem showed TDP-43 type A pathology but with an unusual novel feature of numerous TAR DNA-binding protein 43 (TDP-43)–positive neuritic structures at the cerebral cortex/subcortical white matter junction. There was also associated argyrophilic grain disease not previously reported in other *TBK1* mutation cases.

**Discussion:**

*TBK1*-associated FTD can be associated with right temporal variant FTD with progressive behavioral change and relatively intact cognition initially. The case further highlights the benefits of next-generation sequencing technologies in the diagnosis of neurodegenerative disorders and the importance of detailed neuropathologic analysis.

## 1 Introduction

Frontotemporal dementia (FTD) is a frequent cause of young-onset dementia with around one-third of cases being familial. Patients may present clinically with behavioral or language problems, with around 10% to 15% also developing motor neurone disease (MND). Mutations in several genes have been linked to FTD and MND with *C9orf72* being the most commonly associated gene. However, recent studies have identified mutations in the TANK-binding kinase 1 (*TBK1*) as a novel cause of both FTD and MND [1], [2], [3], [4], [5]. Patients with *TBK1* mutations have been described with the clinical syndrome of FTD (usually the behavioral variant), MND (usually amyotrophic lateral sclerosis), or the combination of both [6], but few details are currently known about the clinical phenotype, atrophy pattern, and time-course of the disease. In this study, we present a longitudinal case report of a patient with a novel *TBK1* mutation assessed over several years.

## 2 Methods

The patient had consented to be part of a longitudinal study at the Dementia Research Centre, UCL Institute of Neurology, approved by the Local Ethics Committee. As part of the study he underwent a standardized clinical history and examination, neuropsychometric testing, and three-dimensional T1-weighted magnetic resonance imaging (MRI), initially on a 1.5GE Signa scanner (first four scans) and then on a 3T Siemens Trio scanner. Using the volumetric MRI, we calculated cortical volumes using an automated segmentation method as previously described [7]. We also manually segmented the caudate, hippocampus, amygdala, and hypothalamus [8], [9], [10]. All brain volumes were corrected for total intracranial volume, which was calculated using SPM12 (www.fil.ion.ucl.ac.uk/spm). We used the SPM12 Serial Longitudinal Registration tool to estimate the percentage of volumetric contraction and expansion for each voxel across the different follow-up visits.

The patient consented to brain donation and after death his brain was assessed using standard pathological methods at the Queen Square Brain Bank for Neurological Disorders, UCL Institute of Neurology. Tissue sections of 7-μm thickness were immunostained using commercially available antibodies to the following proteins: TDP-43 (1:800; 2E2-D3; Abnova); p62 (1:200; BD Transduction Laboratories, Oxford, UK); ubiquitin (1:200; Dako, Ely, UK); α-synuclein (1:1000; 42/syn; BD Biosciences), tau (1:600; AT8; Thermo), or Aβ (1:100; 6F/3D; DAKO) as previously described [11]. Briefly, immunohistochemistry for all antibodies required pressure cooker pretreatment in citrate buffer, pH 6.0. Endogenous peroxidase activity was blocked with 0.3% H_2_O_2_ in methanol and nonspecific binding with 10% dried milk solution. Tissue sections were incubated with the primary antibodies, followed by biotinylated anti-mouse immunoglobulin G (1:200, 30 minutes; DAKO) and ABC complex (30 minutes; DAKO). Color was developed with diaminobenzidine/H_2_O_2_.

The patient was tested for mutations in the *C9orf72* gene and also using a panel examining 17 genes linked to neurodegeneration (*APP*, *CHMP2B*, *CSF1R*, *FUS*, *GRN*, *ITM2B*, *MAPT*, *NOTCH3*, *PRNP*, *PSEN1*, *PSEN2*, *SERPINI1*, *SQSTM1*, *TARDBP*, *TREM2*, *TYROBP*, and *VCP*) [12]. These were negative. Whole-exome sequencing was subsequently carried out using the Agilent SureSelect Human All Exon v2 target enrichment kit (Agilent, Santa Clara, CA) followed by paired-end sequencing performed on an Illumina HiSeq2000 (Illumina, San Diego, CA), achieving an average 30-fold depth-of-coverage of target sequence. After trimming and quality control, sequencing reads were aligned to the human reference genome (hg19) using Burrows-Wheeler Aligner [13], followed by variant calling and recalibration by Genome Analysis Toolkit [14] and annotation with SnpEff [15]. Variants of interest were validated by Sanger sequencing. This revealed a novel 13 base pair deletion causing a frameshift mutation in the *TBK1* gene (c.G2114del-CTGAAAATAACCA; p.A705fs).

## 3 Results

A retired right-handed gentleman presented at the age of 64 years with an 18-month history of personality change including increased rigidity and obsessiveness, apathy, loss of empathy, and development of a sweet tooth. His mother had developed progressive behavioral and cognitive impairment from the age of 57 years but without a formal diagnosis. At his first assessment his Mini-Mental State Examination was 30/30 and neuropsychometry revealed intact cognition (Table 1). Over the next few years his behavioral problems progressed with worsening of his initial symptoms and the development of disinhibition. His cognition also started to become impaired: by 2 years after his initial visit he had developed anomia and prosopagnosia, with impairment on tests of naming and face memory (Table 1). Over the next few visits he subsequently developed impairment of executive function and verbal episodic memory, followed by visuoperceptual problems, and finally impaired single word comprehension and dyscalculia (Table 1). Neurological examination remained normal throughout without any features of MND. He died at the age of 72 years after 9 years of illness.

**Fig. 1:**
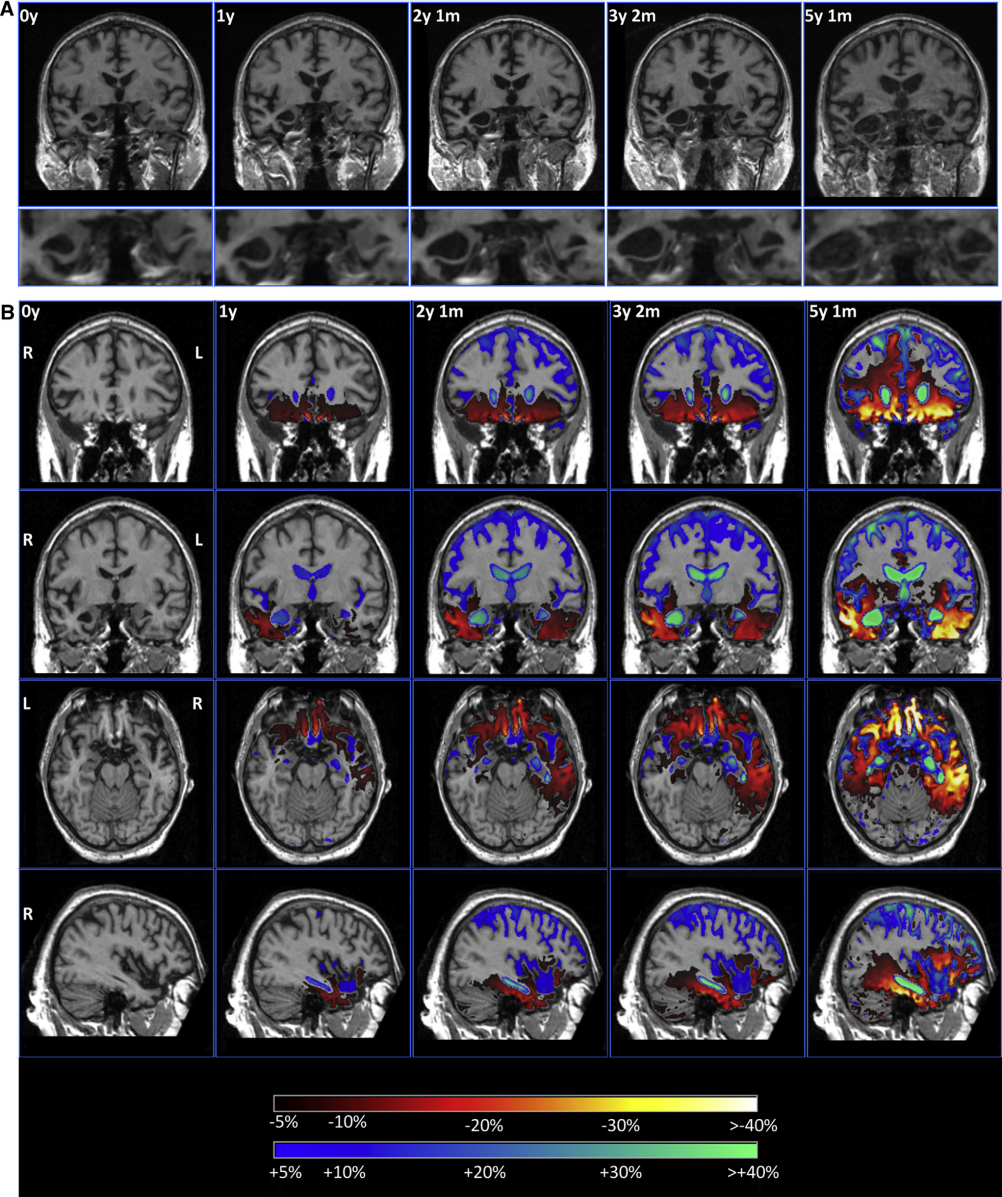
(A) Coronal sections of T1 volumetric magnetic resonance imaging scans at baseline and at the following four follow-up visits. In the lower panels, a close-up of the amygdala shows progressive asymmetrical medial temporal lobe atrophy. (B) Coronal sections at the level of the frontal (top) and mid-temporal lobes (second row), axial section through the orbitofrontal and medial temporal lobe (third row), and sagittal section through the right hemisphere (bottom). The first column represents the baseline scan with the four columns to the right showing a longitudinal SPM overlay (in comparison with the baseline scan) with yellow/red representing 5% or greater volumetric contraction and blue representing 5% or greater volumetric expansion. At the first follow-up scan atrophy is localized to the right temporal lobe and orbitofrontal lobe, but over time spreads dorsally and posteriorly, involving the frontal lobe, posterior temporal lobe, and similar areas in the contralateral hemisphere.

His initial MRI scan showed evidence of focal right temporal lobe atrophy, particularly affecting the anterior temporal lobe and the amygdala (Table 1, Fig. 1). Over a period of time, atrophy spread from the right temporal lobe to involve the right frontal lobe (particularly the orbitofrontal cortex), and more posteriorly to involve the right posterior temporal lobe, hippocampus, and anterior parietal lobe. Subcortical involvement including the caudate and hypothalamus was also seen (Table 1). Atrophy progressed over time to involve the left hemisphere, following a similar pattern to the right, focused initially on the anterior and medial temporal lobe (Fig. 1). However, atrophy remained asymmetrical throughout the disease process, being greater on the right than on the left side (Table 1, Fig. 1).

**Fig. 2:**
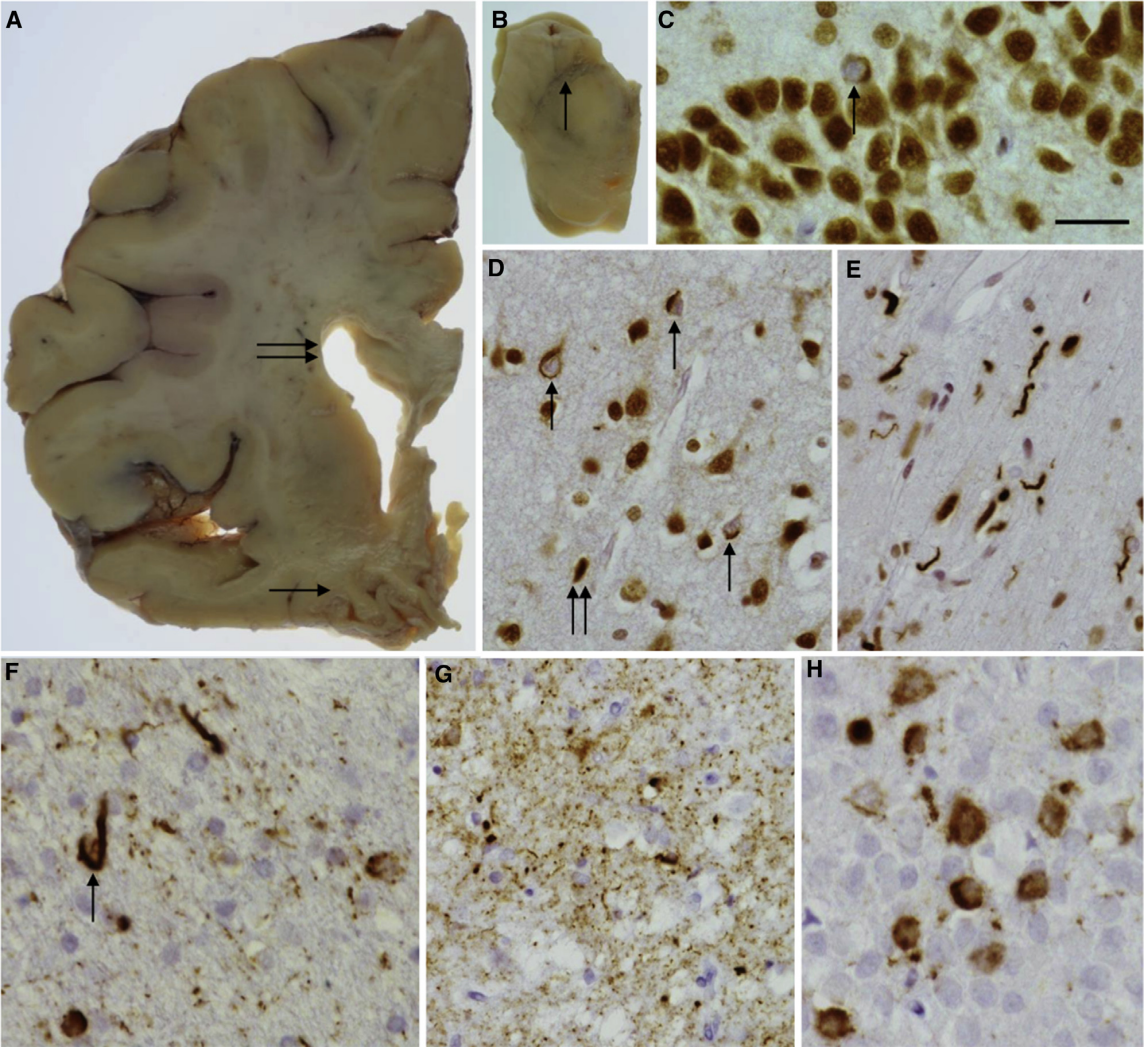
Macroscopic and microscopic pathological features. Enlargement of the lateral ventricle was evident (A, double arrow) and severe atrophy of the temporal lobe (A, arrow). The substantia nigra showed severe pallor (B, arrow). TDP-43 immunohistochemistry highlighted occasional neuronal cytoplasmic inclusions in the granule cell layer of the hippocampus (C, arrow). Neuronal cytoplasmic inclusions (D, arrow) and short neuropil threads (D, double arrow) were also observed in the gray matter of the temporal cortex and many short curved dystrophic neurites were seen in the white matter (E). Tau immunohistochemistry showed coiled bodies in the temporal white matter (F, arrow), argyrophilic grains in the subiculum (G), and neurofibrillary tangles in the granule cell layer of the hippocampus (H). Bar in C represents 40 μm in C, E, and H and 60 μm in D, F, and G.

Macroscopic examination of the brain revealed cortical atrophy, which was more severe in the temporal lobe than the frontal lobe (Fig. 2A) with severe reduction in the bulk of the amygdala and hippocampus, and evidence of hippocampal sclerosis. The substantia nigra showed severe pallor (Fig. 2B), and the locus coeruleus was also pale. The brainstem and cerebellum appeared normal. Microscopically, the normal neocortical hexalaminar architecture was variably disrupted by spongiosis and nerve cell loss across the cortex. This was mild atrophy in the prefrontal cortex but severe in the anterior cingulate gyrus and very severe in the temporal cortex with a clear gradient toward the medial temporal lobe structures. Accordingly, the fusiform gyrus and the parahippocampus were the most severely affected regions showing severe nerve cell loss and an advanced spongy state. α-Synuclein and Aβ immunohistochemical preparations were negative throughout all areas and no “star-like” p62-positive granular inclusions were seen in the granule cells of the dentate fascia or the cerebellum. TDP-43 immunohistochemistry demonstrated a mixture of different lesions in the cortical areas, including neuronal cytoplasmic inclusions, short curved dystrophic neurites, and coiled bodies (Fig. 2D). An unusual feature was the presence of numerous TDP-43–positive neuritic structures and also oligodendroglial inclusions at the cerebral cortex/subcortical white matter junction (Fig. 2E). Only occasional TDP-43–positive neuronal cytoplasmic inclusions were seen in the granule cells of the dentate fascia (Fig. 2C) and only occasional neuritic structures in the amygdala. TDP-43–positive inclusions corresponded to frontotemporal lobar degeneration (FTLD)-TDP type A pathology. Lower motor neurons were investigated in the 12th nerve nucleus for TDP-43 pathology: no pathological inclusions were seen and normal TDP-43 immunohistochemistry was observed with TDP-43 found in the nucleus, i.e. there was no pathological evidence of MND. Tau immunohistochemistry demonstrated not only occasional neuropil threads (NTs) in the prefrontal and parietal cortices but also neurofibrillary tangles, pretangles, NTs, and coiled bodies (Fig. 2F) in the temporal cortex, particularly in the medial temporal region. Severe tau pathology was observed in the hippocampal formation and parahippocampus, where pretangles and NTs were seen throughout and grain-like structures observed in the subiculum (Fig. 2G) consistent with argyrophilic grain disease. Pretangles and neurofibrillary tangles were seen in the granule cells of the dentate fascia (Fig. 2H). Tau-positive coiled bodies, NTs, and an occasional tufted astrocyte-like structure were seen in the pontine tegmentum. There was minimal Purkinje cell loss in the cerebellar cortex and occasional NTs were present in the dentate nucleus.

## 4 Discussion

We describe the clinical, cognitive, and neuroanatomical progression over 9 years of a patient with FTD due to a novel *TBK1* mutation who was found to have FTLD-TDP type A pathology. This case highlights a number of important and novel aspects of *TBK1* mutations as well as the description of a novel mutation, it also reveals the clinical and neuroanatomical phenotype that can be associated with *TBK1* mutations, and the nature of disease progression.

The 13 base pair deletion causes a frameshift introducing a premature stop codon and is therefore likely to be pathogenic even in the absence of supporting segregation or functional data. Haploinsufficiency is a known disease mechanism for *TBK1*, and loss-of-function mutations in *TBK1* have already been described in patients with FTD, MND, and FTD-MND [1], [2], [3], [4], [5].

The most common clinical phenotype associated with *TBK1* mutations is FTD-MND rather than FTD alone, with the frequency in patient cohorts 3.0% to 4.5% and 0.5% to 1.1%, respectively [3], [4]. However, despite close clinical follow-up, our patient exhibited no signs of MND. In terms of the specific FTD phenotype, most patients described in the literature so far have presented with behavioral variant FTD (bvFTD), rather than the language variant, consistent with the case here, although there appears to be a high prevalence of early memory impairment [3], [6]. Despite our patient complaining of subjective cognitive symptoms early in the disease, his neuropsychometric testing remained normal initially with the development of anomia and prosopagnosia only a few years into the illness. The disease duration in our patient was 9 years with an onset at 63 years, which is not dissimilar from findings of a recent study showing a mean onset of 66.3 years and a disease duration of 8.2 years [6].

Previous studies have not detailed the neuroanatomical phenotype of *TBK1* mutations. Here, we have shown an association with right temporal lobe atrophy. This anatomical variant of FTD has been found to be caused by a number of pathologies, with patients presenting with bvFTD more likely to have tau pathology, and those with the semantic dementia phenotype likely to have TDP-43 pathology [16], [17]. Here, we describe a case with bvFTD who has FTLD-TDP type A and a *TBK1* mutation, a novel association with right temporal lobe variant FTD. Progression of atrophy over time was consistent with a number of other right temporal lobe cases who remain with an asymmetrical pattern of involvement but develop a similar pattern of focal temporal lobe involvement in the opposite temporal lobe as the disease progresses.

Little is known about the neuropathology of *TBK1* mutations but previous cases have shown either FTLD-TDP type A or type B pathology, with the histological findings in our case being consistent with type A. Although it fits criteria for this subtype, there were nonetheless some unusual novel features with the presence of numerous TDP-43–positive neuritic structures at the cerebral cortex/subcortical white matter junction. This case also had associated tau pathology consistent with argyrophilic grain disease, which has not been seen in other *TBK1* mutation cases.

Further research is needed to understand the complete phenotype of patients with *TBK1* mutations but it should be considered as a cause of right temporal variant FTD, and in patients who present with familial FTD, whether MND is present or not.

**Research in context**

1. Systematic review: TBK1 mutations have recently been linked to frontotemporal dementia (FTD). To put our findings into context, we reviewed the literature on Pubmed Central relating to other published *TBK1* cases and research.
2. Interpretation: Our findings detail the natural long-term progression in a case of *TBK1*-associated FTD and further characterize the related neuropathologic findings. In addition, the case emphasizes the benefits of next-generation sequencing technologies in the diagnosis of neurodegenerative disorders.
3. Future directions: Further functional studies of *TBK1* and other genes linked to neurodegenerative diseases are needed to fully comprehend how loss-of-function and other mutations affect cellular pathways and cause disease.

## Figures and Tables

**Table 1:** Longitudinal neuropsychometric and neuroimaging measures

Cognitive and imaging measures	Initial visit	1 y	2 y 1 m	3 y 2 m	4 y 11 m∗	5 y 11 m	6 y 11 m
Neuropsychometry							
Mini-Mental State Examination	30	30	30	28	24	NT	23
Wechsler Abbreviated Scale of Intelligence Total IQ score	NT	NT	≥75%–95%	≥75%–95%	≥25%–50%	≥25%–50%	≥5%–10%
Episodic memory							
Recognition Memory Test for Words	≥95%	≥95%	≥75%	NT	≥10%–25%	<5%	<5%
Recognition Memory Test for Faces	≥50%–75%	≥50%	≥5%–10%	NT	<5%	<5%	<5%
California Verbal Learning Test Delay Recall	≥75%	≥75%–95%	≥50%	NT	NT	NT	NT
California Verbal Learning Test Delay Recognition	≥75%–95%	≥75%–95%	≥75%–90%	NT	NT	NT	NT
Language							
Graded Naming Test	≥50%–75%	≥50%–75%	≥5%–10%	NT	<5%	<5%	<5%
British Picture Vocabulary Scale	NT	NT	NT	NT	≥50%–75%	<5%	<5%
Synonyms Task	NT	NT	≥95%	≥10%–25%	NT	NT	NT
National Adult Reading Test	≥75%–95%	≥75%–95%	≥75%–95%	NT	≥75%–95%	≥25%–50%	≥25%–50%
Calculation							
Graded Difficulty Arithmetic Test	NT	NT	NT	≥95%	≥95%	≥95%	≥75%–95%
Visuoperceptual skills							
Visual Object and Space Perception battery: Object Decision subtest	NT	NT	≥75%–95%	≥50%–75%	≥25%–50%	≥5%–10%	<5%
Attention and executive function							
D-KEFS Color-Word Interference Test							
Color Naming	NT	NT	NT	NT	≥25%–50%	<5%	<5%
Word Reading	NT	NT	NT	NT	≥50%–75%	≥25%–50%	≥50%–75%
Ink-Color Naming	NT	NT	NT	NT	≥50%–75%	<5%	≥25%–50%
Trail Making Test Part A	≥10%–25%	≥10%–25%	≥10%–25%	≥5%–10%	NT	NT	NT
Trail Making Test Part B	≥10%–25%	≥50%–75%	≥25%–50%	≥25%–50%	NT	NT	NT
Wechsler Memory Scale-Revised Digit Span Forward	NT	NT	NT	NT	≥50%–75%	≥25%–50%	≥25%–50%
Wechsler Memory Scale-Revised Digit Span Backward	NT	NT	NT	NT	≥50%–75%	≥50%–75%	≥50%–75%
Neuroimaging							
Cortical volumes (% of TIV)							
Frontal							
Right	5.3	5.2	5.3	5.1	4.9	NA	NA
Left	5.5	5.5	5.5	5.4	5.2	NA	NA
Temporal							
Right	3.2	3.1	2.9	2.8	2.8	NA	NA
Left	3.7	3.6	3.5	3.3	3.2	NA	NA
Insula							
Right	0.33	0.29	0.28	0.26	0.26	NA	NA
Left	0.38	0.36	0.33	0.30	0.31	NA	NA
Subcortical volumes (% of TIV)							
Caudate							
Right	0.17	0.16	0.15	0.12	0.10	NA	NA
Left	0.17	0.18	0.16	0.15	0.13	NA	NA
Hippocampus							
Right	0.13	0.13	0.12	0.10	0.09	NA	NA
Left	0.13	0.13	0.12	0.11	0.10	NA	NA
Amygdala							
Right	0.056	0.056	0.040	0.041	0.032	NA	NA
Left	0.069	0.064	0.052	0.047	0.039	NA	NA
Hypothalamus							
Right	0.029	0.021	0.019	0.016	0.016	NA	NA
Left	0.030	0.023	0.024	0.019	0.017	NA	NA

Abbreviations: NA, not analyzable; NT, not tested; D-KEFS, Delis-Kaplan Executive Function System; TIV, total intracranial volume.

NOTE. For neuropsychometric measures, scores are given as percentiles. For neuroimaging measures, volumes of regions are given as a percentage of TIV.

∗ For fifth visit, neuropsychometry was performed at 4 years 11 months from baseline and magnetic resonance imaging scan was performed at 5 years 1 month.
